# Sleep Quality is Poor in Rheumatoid Arthritis Patients and Correlates with Anxiety, Depression, and Poor Quality of Life

**DOI:** 10.31138/mjr.221022.sqp

**Published:** 2024-02-07

**Authors:** Gurmeet Singh, Vijay Kumar

**Affiliations:** Department of Medicine, Government Medical College Jammu, Jammu and Kashmir, India

**Keywords:** rheumatoid arthritis, sleep, anxiety, depression, quality of life

## Abstract

**Objective:**

Sleep quality is poor in most rheumatoid arthritis (RA) patients. We planned this study to see the association of sleep quality with anxiety, depression, and quality of life (QOL) in Indian RA patients.

**Methods:**

One hundred twelve RA patients and 93 controls were included in this cross-sectional study. Sleep quality was assessed by Pittsburgh Sleep Quality Index (PSQI). Quality of life was assessed using the World Health Organisation Quality of Life (WHOQOL-BREF). Anxiety and depression were assessed using the hospital anxiety and depression scale (HADS). The disease activity was measured by Disease Activity Score for 28 joints with 3 variables. Functional disability was measured by using the Indian version of the Health Assessment Questionnaire (HAQ).

**Results:**

Poor sleep quality was seen in 103(92%) of the patients and 26(28%) controls (p<0.0001). RA patients had significantly higher PSQI scores as compared to the control group (10.8±3.7 vs.3.9±1.2, P<0.0001). Poor sleepers were more likely to be females (90% vs.0%, p = 0.02), and had longer disease duration (5.2±4.8 years vs. 2.8±2 years, p=0.01). Poor sleepers had more pain (VAS 42.2±23.6 mm vs.16.2±11.6 mm, p<0.001) and poor functional status (HAQ 1.2±0.5 vs. 0.6±0.4, p<0.01). There was a significant effect of the physical domain of WHOQOL-BREF, anxiety, and age on poor sleep quality.

**Conclusion:**

Poor sleep quality is seen in a majority of RA patients and correlates with anxiety, depression, and poor QOL. RA patients need to be routinely assessed for sleep quality and factors affecting poor sleep quality need to be addressed.

## INTRODUCTION

Rheumatoid arthritis (RA) is a chronic inflammatory arthritis of autoimmune origin affecting around 0.5–1% of the population.^1^ Sleep disturbance is one of the important predictors of quality of life in RA patients and it has been identified as an important contributor to poor QOL by RA patients.^2^ Sleep disturbance may be because of multiple factors in RA such as inflammation, joint pain, fatigue, medications, or other co-morbidities like fibromyalgia.^3^ Disturbed sleep may not only affect the QOL but may also have an impact on the psychological and social well-being of the patients, flare of disease activity, increased general and mental fatigue, and daytime sleepiness.^2–4^ A recent study reported that people with poor sleep quality and decreased sleep duration had higher odds of developing RA so the relationship between sleep and RA might be bidirectional.^5^ Poor sleep quality may also affect functional disability through its relationship with pain severity and fatigue.^6^ A recent study from India reported a high prevalence of poor sleep quality in RA patients and found that high risk of sleep apnoea, fatigue, and physical health domain of WHOQOL-BREF were independent predictors of poor sleep.^7^ Depression and anxiety may affect the sleep quality in RA patients and these patients may require psychological interventions targeted to improve underlying psychological morbidity.^8^ Depression may independently contribute to sleep disturbances. Depression may act as a significant mechanism through which pain contributes to sleep disturbance.^9^ A Chinese study reported significant correlations among pain, disease activity, anxiety, depression, and sleep quality in RA patients.^10^ Pain, fatigue, and depression may act in conjunction to have direct or indirect impact on sleep disturbance.^11^ Depression affects the RA patient in multiple ways. Patients with underlying depression are more likely to have poor QOL, poor compliance with medications, decreased pain threshold, and increased pain perception. Depression in RA is also associated with poor sleep and fatigue.^12^ As there is a paucity of data regarding the relationship between sleep quality and anxiety, depression, and quality of life in RA patients from India, we planned this study to see the prevalence of sleep quality in RA patients as compared to the control population and its relationship with psychological morbidity in the form of anxiety, depression, quality of life, and clinical parameters in RA patients.

## METHODS

The present study was conducted in a tertiary care teaching hospital in North India. Patients with RA as per 2010 ACR-EULAR classification criteria aged more than 16 years were included in the study.^13^ Patients with co-morbidities such as diabetes, chronic kidney disease, chronic obstructive pulmonary disease, coronary artery disease, congestive heart failure, rheumatic heart disease, and inflammatory bowel disease were excluded from the study. Pregnant and lactating mothers and patients with fibromyalgia were also excluded from the study. Consecutive patients were recruited over a period of one year from October 2015 to November 2016. Age and sex-matched control were recruited from the accompanying relatives of the patients and hospital staff. Sleep quality was examined by Pittsburgh Sleep Quality Index (PSQI), which includes 19 questions answered by the subject and covers seven components, i.e. subjective sleep quality, sleep latency, sleep duration, habitual sleep efficiency, sleep disturbances, use of sleeping medications, and daytime dysfunction over the last month. It differentiates poor from good sleep quality and a score of five or more on this scale indicates poor sleep quality while a score of four or less reflects good sleep quality.^14^ Quality of life was assessed by using the World Health Organisation Quality of Life (WHOQOL-BREF) Hindi version, which is a patient-administered 26-item questionnaire with a possible score of 1- 5 for each question.^15,16^ This questionnaire assesses the scores in four domains, i.e. physical, psychological, social relationships, and environment, with higher scores reflecting better QOL.

The physical health domain covers activities of daily living, mobility, pain, discomfort sleep, rest, and work capacity. It includes questions such as:

- Do you have enough energy for everyday life?
- How well are you able to get around?
- How satisfied are you with your ability to perform your daily living activities?

The psychological domain covers body image and appearance, negative feelings, positive feelings, and self-esteem with questions such as:

- How much do you enjoy life?
- To what extent do you feel your life to be meaningful?
- Are you able to accept your bodily appearance?
- How satisfied are you with yourself?
- How often do you have negative feelings such as blue mood, despair, anxiety, or depression?

The social domain includes personal relationships, social support, and sexual activity with questions such as:

- How satisfied are you with your personal relationships?
- How satisfied are you with your sex life?
- How satisfied are you with the support you get from your friends?

And the environmental domain covers financial resources, freedom, physical safety and security, health and social care: accessibility and quality, home environment, participation in and opportunities for recreation/leisure activities, and transport. The questions in this domain include:

- How safe do you feel in your daily life?
- How healthy is your physical environment?
- Do you have enough money to meet your needs?
- To what extent do you have the opportunity for leisure activities?
- How satisfied are you with your access to health services?
- How satisfied are you with your transport?

Each item of the WHOQOL-BREF is scored from one to five on a five-point ordinal scale. The scores for each domain are added separately and transformed linearly to a 0–100 scale. Hospital anxiety and depression scale (HADS) was used to assess the symptoms of anxiety and depression. HADS has seven questions each for anxiety and depression with a maximum possible score of three for each question. A score of 0–7 is considered normal, 8–10 as borderline abnormal, and 11–21 as abnormal.^17^ The disease activity was measured by Disease Activity Score for 28 joints (DAS28) with 3 variables, i.e. swollen joint count (SJC), tender joint count (TJC), and erythrocyte sedimentation rate (ESR) by Westergren’s method. Higher scores indicate higher disease activity.^18^ Functional disability was measured by using the Indian version of the Health Assessment Questionnaire (HAQ), with higher scores indicating more disability.^19^ The severity of pain was assessed using a 100 mm visual analog scale (VAS), with 0 representing no pain and 100 representing maximum possible pain.^20^

The data were analysed using OpenStat software. Baseline characteristics as well as the PSQI component scores of the patients and controls were compared. Also, demographic and clinical parameters of the poor and good sleepers were compared. The means of continuous variables were compared by Student’s “t” test, and the categorical variables were compared by Fisher’s exact test. A p-value of <0.05 was considered significant. Pearson’s correlation coefficient was calculated between PSQI scores and age, disease duration, anxiety, depression, WHOQOL domains, HAQ, DAS, and pain VAS. For correlations P<0.01 was considered statistically significant. The best fit Multiple regression model was used to see the effect of age, disease duration, DAS 28, pain (VAS), HAQ, anxiety (HADS-A), depression (HADS-D), physical, psychological, social relationships, and environment domains of WHOQOL-BREF on the sleep quality (PSQI). The demographic and clinical variables were taken as independent variables while the PSQI score was taken as dependent variable. Study protocol was approved by Institutional Ethics Committee vide orders IEC/Thesis/Research/TB/C-36/2015/232. Informed consent obtained from all the patients and controls.

## RESULTS

A total of 112 RA patients and 93 controls were included in the study, 103(92%) patients had poor quality sleep as compared to 26 (28%) in the control group (p<0.0001). There was no significant difference in the age, sex, and marital status of the patients and controls **(Table 1)**. RA patients had higher PSQI scores as compared to the control group (10.8±3.7 vs.3.9±1.2, P<0.0001) reflecting disturbed sleep. RA patients had significantly more anxiety and depression as compared to the healthy controls. Similarly, RA patients had significantly lower scores in all the domains of WHOQOL i.e. physical health, psychological, social, and environmental domains as compared to the control group **(Table 1)**. Patients with RA had higher scores in all the components of PSQI when compared to controls **(Table 2)**.

**Table 1.** Baseline characteristics of RA patients and controls.

**Variable**	**Patients (112)**	**Control (93)**	**p-value**
Age (years)	46.8±11.8	45.4±9.8	NS
Females (%)	93(83)	74(79)	NS
Married (%)	106(94)	84(90)	NS
PSQI Score	10.8±3.7	3.9±1.2	<0.0001
Anxiety score	8.3±3.4	0.7±1.0	<0.0001
Depression score	7.4±3.9	0.2±0.6	<0.0001
Physical Health	40.9±15.6	82.7±5.0	<0.0001
Psychological	57.6±13.6	78.0±6.1	<0.0001
Social	62.4±13.5	77.4±7.4	<0.0001
Environmental	56.4±9.8	71.1±7.4	<0.0001
Disease duration (years)	5.0±4.7		
DAS 28-3	4.7±1.2		
HAQ	1.1±0.5		
Pain VAS (mm)	40.1±23.9		

PSQI: Pittsburgh Sleep Quality Index; DAS28-3: Disease Activity Score; HAQ: Health Assessment Questionnaire; VAS: Visual Analogue Scale; NS: Not significant.

Values indicate M±SD: Mean±Standard deviation.

**Table 2.** Comparison of PSQI components of RA patients and controls.

**PSQI components score**	**Patients (112) mean±SD**	**Control (93)**	**P**
Subjective sleep quality	1.61±0.53	0.89±0.31	<0.0001
Sleep latency-questions	2.30±0.85	0.87±0.54	<0.0001
Seep duration	1.77±0.67	0.95±0.71	<0.0001
Habitual sleep efficiency	1.54±1.15	0.23±0.45	<0.0001
Sleep disturbances	1.45±0.50	0.95±0.23	<0.0001
Use of sleep-promoting medications	1.04±1.38	0.09±0.28	<0.0001
Daytime dysfunction	1.13±0.70	0.03±0.18	<0.0001
Total	10.82±3.79	3.91±1.22	<0.0001

PSQI: Pittsburgh Sleep Quality Index; SD: Standard deviation.

### Comparison of poor and good sleepers

Most of the patients (92%) were poor sleepers. There was no significant difference in the age, DAS, and environmental domains between poor and good sleepers. However poor sleepers were more likely to be females (90% vs.0%, p = 0.02), and had longer disease duration (5.2±4.8years vs. 2.8±2 years, p=0.01). Poor sleepers also had more pain (VAS 42.2±23.6 mm vs.16.2±11.6 mm, p<0.001) and poor functional status (HAQ 1.2±0.5 vs. 0.6±0.4, p<0.01). Poor sleepers had more anxiety, depression, and poor quality of life in all the domains of WHOQOL- BREF except in the environmental domain **(Table 3)**.

**Table 3.** Comparison of poor and good sleepers.

**Variable**	**Poor sleepers (103)**	**Good sleeper (9)**	**P**
Age(years)	47±11.6	45±14.6	NS
Females (%)	93(90)	0 (0)	0.02
Married (%)	103(100)	3(33)	NS
PSQI Score	11.3±3.4	3.8±0.3	<0.001
Anxiety	8.7±3.2	3.7±2.7	<0.001
Depression	7.9±3.7	2.7±2.8	<0.001
Physical Health	38.9±14.2	64.6±11.2	<0.001
Psychological	56.5±13.4	70.1±8.2	<0.01
Social	61.5±13.3	72.8±11.4	<0.05
Environmental	56.1±9.4	59.8±13.2	NS
Disease duration(years)	5.2± 4.8	2.8±2.0	NS
DAS 28-3	4.8±1.2	4.1±1.1	NS
HAQ	1.2±0.5	0.6±0.4	<0.01
Pain VAS (mm)	42.2±23.6	16.2±11.6	<0.001

DAS28-3: Disease Activity Score; HAQ: Health Assessment Questionnaire; VAS: Visual Analogue Scale; NS: Not significant; SD: Standard deviation.

Values indicate M±SD: Mean±Standard deviation

### Correlation between clinical variables and components of PSQI

Poor sleep quality had a significant positive correlation with age, disease duration, anxiety, depression, HAQ, DAS, and VAS. There was a significant negative correlation between poor sleep quality with all the domains of WHOQOL-BREF **(Table 4)**. There was also significant positive correlation of anxiety, depression, HAQ, DAS, and VAS with all the components of PSQI except the use of the sleep medication component. Similarly, there was a significant negative correlation between all the domains of WHOQOL-BREF with all the components of PSQI except the use of the sleep medication component **(Table 4)**. Multiple regression analysis of demographic, medical, and psychological parameters as independent variables with PSQI as dependent variable revealed a significant effect of the physical domain of WHOQOL-BREF, anxiety, and age on sleep quality **(Table 5)**.

**Table 4.** Correlation between clinical variables and components of PSQI.

**Variable**	**Subjective sleep quality**	**Sleep latency**	**Sleep duration**	**Habitual sleep efficiency**	**Sleep disorders**	**Use of sleep medication**	**Daytime dysfunction**	**Global PSQI**
Age	0.12	0.18	0.18	0.16	0.18	0.03	0.21*	0.20*
Disease duration	0.15	0.19*	0.00	0.28**	0.25**	0.09	0.23*	0.26**
Anxiety	0.53***	0.58***	0.37***	0.61***	0.48***	0.11	0.56***	0.67***
Depression	0.56***	0.63***	0.30***	0.61***	0.45***	0.14	0.63***	0.69***
Physical health	−0.62***	−0.69***	−0.32***	−0.67***	−0.52***	−0.13	−0.60***	−0.73***
Psychological health	−0.55***	−0.55***	−0.26**	−0.59***	−0.38***	−0.17	−0.56***	−0.64***
Social relations	−0.46***	−0.39***	−0.01	−0.37***	−0.39***	−0.05	−0.36***	−0.40***
Environmental health	−0.36***	−0.41***	−0.1187	−0.42***	−0.38***	0.01	−0.30***	−0.40***
HAQ	0.43***	0.50***	0.23*	0.47***	0.43***	0.07	0.53***	0.54***
DAS	0.29**	0.31**	0.07	0.31***	0.25**	−0.11	0.37***	0.294**
VAS	0.35***	0.47***	0.22*	0.39***	0.25**	−0.03	0.46***	0.41***

* P<0.05,

** P<0.01,

*** P<0.001, PSQI: Pittsburgh Sleep Quality Index; DAS: Disease Activity Score; HAQ: Health Assessment Questionnaire; VAS: Visual Analogue Scale.

**Table 5.** Multiple regression analysis for the effects of demographic, medical, and psychological parameters on PSQI.

**Variable**	**Beta**	**B**	**S E**	**P**
Age	0.143	0.046	0.020	0.02
Anxiety	0.269	0.292	0.105	<0.01
PHYSICAL	−0.520	−0.126	0.023	<0.001

PSQI: Pittsburgh Sleep Quality Index.

B: Unstandardised regression coefficients; β: Standardised regression coefficients; SE: standard error of the Unstandardised coefficient.

Significance at P value <0.05.

### Comparison of sleep quality in patients with and without anxiety and depression

We divided the patients into two groups as having anxiety and depression keeping the cut-off score at ≥ 11. Patients with anxiety had significantly poor quality of sleep in all the domains of PSQI except “use of sleep-promoting medication” domain when compared to patients without anxiety. Similarly, patients with depression had significantly poor sleep quality in all the domains of PSQI when compared to the patients without depression **(Table 6)**.

**Table 6.** Comparison of PSQI components of patients with and without anxiety and depression symptoms (HADS Score≥11).

**PSQI components score**	**Anxiety (26) Mean±SD**	**No Anxiety (86) Mean±SD**	**P**	**Depression (24) Mean±SD**	**No Depression (88) Mean±SD**	**P**
Subjective sleep quality	2.08±0.27	1.47±0.50	<0.0001	2.00 ±0.29	1.50± 0.53	<0.0001
Sleep latency-questions	2.96 ±0.20	2.06±0.92	<0.0001	2.58±0.72	2.09±0.88	<0.0001
Seep duration	2.19±0.57	1.60 ±0.67	<0.001	2.00 ±0.66	1.67±0.69	<0.05
Habitual sleep efficiency	2.50±0.81	1.26±1.08	<0.0001	2.54 ±0.66	1.27±1.10	<0.0001
Sleep disturbances	1.81±0.40	1.34 ±0.48	<0.0001	1.71± 0.46	1.38±0.49	<0.01
Use of sleep-promoting medications	1.31±1.49	0.97±1.34	NS	1.83±1.43	0.83 ±1.29	<0.01
Daytime dysfunction	1.69± 0.62	0.97± 0.64	<0.0001	1.67±0.56	0.99± 0.67	<0.0001
TOTAL	14.54±2.00	9.64±3.56	<0.0001	14.75±1.59	9.69±3.58	<0.0001

PSQI: Pittsburgh Sleep Quality Index; SD: Standard deviation; NS: Not Significant; HADS: Hospital anxiety and Depression Scale.

## DISCUSSION

In our study patients with RA had poor sleep quality when compared to controls. Poor sleep quality was seen in 92% of RA patients as compared to 28% in controls. This number appears to be higher when compared to other studies from India (63%) and China (78%).^7,21^ The reason for the lower prevalence of poor quality of sleep in the Indian study could be that patients were enrolled in a private corporate hospital. The patients reporting to such hospitals usually come from a better socioeconomic status as was seen in this study with 65% of patients coming from the upper and upper middle class. Patients from better socioeconomic status are likely to cope better with their disease and its complications as they have better access to health care services, better transport services, and domestic help for household work. The Chinese study had a prevalence of poor sleep quality in 76% as compared to 92% patients in our study. The reason could be lower disease activity (DAS scores 3.96±1.44 vs. 4.7±1.2) and lower disability (HAQ scores 0.50 ± 0.61 vs. 1.1±0.5) in the Chinese study as compared to our study. It has been seen that both high disease activity and high functional disability have an association with poor sleep quality.^7,21^ Also, the patients in the Chinese study had longer disease duration as compared to our patients (8.7 ± 9.1years vs. 1.3±0.4 years). The patients with longer disease duration likely have time to adapt and accept their disease which may lead to these patients having less disturbance of sleep.

Patients of RA had more anxiety, depression, and poor quality of life in all the domains as compared to the control population. This has been seen in various studies.^22–26^ We found that both anxiety and depression are strongly correlated with poor sleep quality. Both anxiety and depression had significant association with poor sleep quality in most of the domains of PSQI **(Table 6)**. Studies have shown that anxiety and depression have a major effect on sleep quality in RA patients.^27^ What could be the source of anxiety in RA patients? A study from Finland on patients with chronic pain suggested that the source of anxiety in these patients could be the pain itself. Anxiety seems to be the physiological response to the pain leading to heightened arousal. This phenomenon of increased arousal occurring at bedtime may make it difficult for the patent to go to sleep.^28^ Depression has a significant correlation with sleep disturbance as seen in various studies.^21,^^29,^^30^ Depression may have a bidirectional relationship with sleep disturbances and sleep disturbance may be a predictive prodromal symptom of depression.^31^ Depression may act as a mechanism through which pain affects sleep.^32^ Depression, pain, and poor sleep may be a triad seen in RA patients with each component affecting the other in a bidirectional manner. A study from United Kingdom reported a close relationship between CRP and depression in RA patients though no relationship was seen between anxiety and CRP. In this study of early RA patients, depression and anxiety were common, and were associated with disease activity and worse functional status and outcome.^33^

Depression may have association with inflammation reflecting as higher disease activity and more pain in patients with depression. In a study from Canada, mindfulness-based stress reduction intervention lead to improvement in anxiety, depression, and sleep and function up to 12 months after the intervention. There was no significant impact on pain and disease activity. The authors suggested that larger studies are required to see the impact of mindfulness-based stress reduction on pain in RA patients.^34^ Does the treatment of RA leads to improvement in sleep? Studies have shown that the treatment of RA leads to improvement in sleep parameters. Treatment with biologicals leads to more pronounced changes in disease activity and sleep improvement.^30–32^ Similar effect of improvement in sleep quality with anti-TNF-α treatment was seen in a group of ankylosing spondylitis patients.^39^ It appears that sleep quality in RA patients is a reflection of multiple factors such as disease duration, disease activity, pain, anxiety, depression, and disability.

There was a significant negative correlation between all the domains of WHOQOL-BREF with sleep quality. The physical domain of WHOQOL-BREF and age also had a significant effect on sleep quality **(Table 5)**. A previous study from India also revealed that the physical domain of the WHOQOL-BREF affected sleep quality.^7^ The Chinese study, on the other hand found that depression and disease activity had a significant effect on sleep quality and sleep quality had a significant association with social function (SF) and Mental Component Summary (MCS) domains of Short Form 36 health survey (SF-36).^21^ Control of disease activity in RA patients have shown to improve the quality of life also.^39^ Poor sleep quality not only impairs the quality of life but also affects the cognitive functions in RA patients.^40^ A meta-analysis also revealed that poor sleep had a negative correlation with physical and mental domains.^41^ A prospective study found that increase in sleep time, decreasing sleep variability, and decreasing sleep onset latency improved the quality of life in RA patients.^42^

Sleep and immunity are linked to each other in a bidirectional manner. Inflammatory response due to infections or inflammatory disorders can disrupt normal sleep. Disturbed sleep on the other hand can aggravate the underlying infection or inflammation by its effect on the immune response.^43^ A study reported evidence of sleep fragmentation, greater sleep depth, and higher levels of cellular inflammation in RA patients as compared to controls. Higher levels of TNF and IL-6 in the evenings were associated with higher sleep efficiency but the relationship between TNF and IL-6 with sleep was reversed in the mornings. According to the authors, this suggests a feedback loop between sleep maintenance, slow-wave sleep, and cellular inflammation.^44^ Treatment of RA patients with anti TNF- alpha therapy has been shown to improve sleep quality.^35^Another study reported improvement in insomnia scores in RA patients treated with Upadacitinib.^45^ Similar improvements in sleep quality after treatment with anti-TNF agents have been seen in patients with inflammatory bowel diseases and ankylosing spondylitis.^37,46^ This suggests the role of inflammatory cytokines and disease activity on sleep quality.

Sleep disturbance appears to be present in a majority of RA patients. Multiple factors like disease activity, disease duration, disability, underlying depression, anxiety, and other co-morbidities affect the sleep quality in RA patients. The relationship between sleep quality and quality of life, anxiety, depression, and disease activity could be bidirectional. Thus, improvement in sleep quality requires assessment of all the above factors and their treatment for optimal management of RA patients.

## LIMITATIONS OF THE STUDY

This was a cross-sectional study; thus, we were able to collect data at a single time point. It has been suggested that depression, pain, and poor sleep may be a triad with each component affecting the other in a bidirectional manner, but we were not able to test this hypothesis as this was a cross-sectional study. The disease activity and other parameters may fluctuate over a time that we may not have been able to capture. The study was conducted in a tertiary care institute. The patients presenting to a tertiary care centre are likely to have more severe disease that may not be reflective of the disease severity seen in primary care.

## CONCLUSION

In our study, we found out that our patients of RA have poor sleep quality as compared to healthy controls. Poor sleep quality correlated with psychological morbidity in the form of anxiety and depression. Poor sleep quality also correlated with a decrease in quality of life. Thus, the patients of RA need to be evaluated for disturbed sleep at each visit. If present, poor sleep quality needs to be addressed to improve the quality of life in these patients. Though there are studies that suggest that control of disease activity leads to improvement of sleep, we need long-term studies to look at the other variables which could be manipulated to improve the sleep quality in RA patients.
